# Different Protecting Groups to Cap Mercapto Propyl Silatrane Affect Water Solubility and Surface Modification Efficiency

**DOI:** 10.1021/acsomega.4c06255

**Published:** 2025-10-01

**Authors:** Wen-Hao Chen, Chih-Yu Chen, Hui-Yin Huang, Yu-Cheng Hsiao

**Affiliations:** a Research and Development Group, Leo Verification Systems Inc., Powell, Wyoming 82435, United States; b Department of Orthopedics, Shuang Ho Hospital, 38032 Taipei Medical University, New Taipei City 23561, Taiwan; c International Ph.D. Program in Biomedical Engineering, College of Biomedical Engineering, 38032 Taipei Medical University, Taipei 11031, Taiwan; d Department of Semiconductor Engineering, Lunghwa University of Science and Technology, Taoyuan 333, Taiwan; e Research and Development Group, Bion Inc., Taipei 110, Taiwan

## Abstract

Surface modification is an important field and widely applied to biosensors, biomaterials, and semiconductors. Mercapto propyl trimethoxyl silane (MPTMS) is a most common material applied to surface modification in biosensor chips. However, MPTMS is moisture sensitive, slow to modify reaction rates with substrate surfaces, and unstable due to thiol groups, which restrict the expansibility of MPTMS. Previously, we synthesized mercapto propyl silatrane (MPS) to improve moisture sensitivity and increase reactivity with substrate surfaces. Despite these improvements, MPS still requires a high-polarity organic solvent environment and the thiol groups remain susceptible to oxidation by oxygen. The utility of mercaptan-functionalized films critically depends on their stability under ambient conditions. As global environmental awareness increases, developing stable and environmentally friendly silane molecules has become increasingly important. In this report, we explored different protective groups (acetyl- (Ac-), di-*tert*-butyl carboxyl- (Boc-), and triphenylmethyl- (trityl-)) to cap the mercapto group of MPS, enhancing the stability of the thiol group. We characterized the Boc-MPS, Ac-MPS, and trityl-MPS modifications on substrates using contact angle measurements, X-ray photoelectron spectroscopy (XPS), and atomic force microscopy (AFM). Additionally, we tracked the kinetic rate of capping MPS modification on substrates by using gold nanoparticles (AuNPs). Our results indicated that all protective groups successfully inhibited the oxidation of the thiol groups. Notably, some protective groups (Ac- and Boc-) increase the water solubility. By tracking the rate of Ac-MPS modification on glass surfaces using AuNPs, we observed that Ac-MPS demonstrated a higher surface modification effectiveness than MPS. Furthermore, the water solubility of Ac-MPS allowed for modification on both glass and plastic substrates in aqueous solutions, significantly broadening the application scope of Ac-MPS for various substrate modifications. In this study, we also tested the LSPR refractive index sensitivity of mercapto propyl trimethoxysilane (MPTMS) and Ac-MPS anchored AuNPs on a glass substrate. The results revealed that glass substrates with Ac-MPS anchored AuNPs exhibited greater sensitivity compared with MPTMS coatings. Interestingly, the refractive index test indicated that plastic substrates with Ac-MPS anchored AuNPs showed better sensitivity than glass substrates. This work presents a novel approach to enhancing the stability, water solubility, and surface coating efficiency of MPS through structural extension. We believe that this method can be widely applied to silatrane extensions, potentially eliminating the need for organic solvents in sol–gel surface modification systems. This work significantly expands the applications of silatrane in green chemistry and sustainable development.

## Graphical abstract

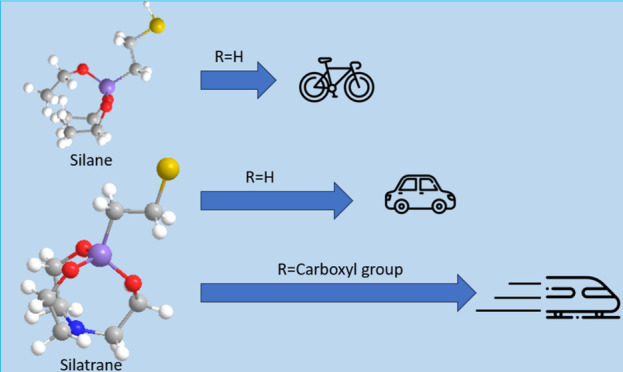

## Introduction

Metal nanoparticles exhibit quantum effects that affect their physical and chemical behavior, making them widely applied in biosensor systems. For example, localized surface plasmon resonance (LSPR) from gold nanoparticles has great sensitivity compared to surface plasmon resonance (SPR) from gold films.^1–3^ Metal nanoparticles also have a hotspot effect and can be widely applied in surface enhanced Raman spectroscopy.^4,5^ Although metal nanoparticles are widely applied to sensor systems, how to uniformly and stably anchor metal nanoparticles is an important issue for researchers. The thiol group can bind with a noble metal by covalent bonding and is popularly used in anchored metal nanoparticles, especially in noble metal nanoparticles.^6–8^ Silane with a mercapto functional group is usually applied to an anchored noble nanoparticle. For example, (3-mercaptopropyl) trimethoxysilane (MPTMS) is often used to construct gold colloid monolayers on silica surfaces for important applications in biosensing, surface-enhanced Raman scattering, catalysis, and so on.^9–12^ MPTMS is commonly applied to create mercapto films on a substrate. Unfortunately, MPTMS is very sensitive to moisture, and this is why MPTMS needs to be modified on a substrate in an organic solvent (like toluene, alcohol, or benzene). However, it is difficult to control the sol gel effect on a substrate, which leads to homogeneity and reproducibility problems.^13–15^

Previously, we successfully synthesized a silatrane structure as mercapto propyl silatrane (H-MPS) to resist the moisture sensitivity of silane. In the silatrane structure, due to a strong intramolecular donor–acceptor interaction between nitrogen and silicon atoms, silatranes are chemically more stable to hydrolysis than trialkoxysilanes.^16,17^ Even the silatrane structure raises the stability of H-MPS in a moisture environment, but H-MPS still needs a high polar organic solvent for modification on a substrate.^15^ Unfortunately, most plastics will be damaged by organic solvents,^18^ and organic solvents have high cell toxicity^19^ and high cost and are not environmentally friendly. Based on these issues, silane or silatrane molecules need work under organic solvents, but mostly limited to silanization of plastic substrate modification. For this reason, we used a protecting group by thiol ester to inhibit the thiol oxidation and also create a hydrogen bond to cap MPS to raise its water solubility. Here, we used the two common protecting groups, acetyl- (Ac-) and di-*tert*-butyl carboxyl- (Boc-), to create a hydrogen bond donor by the carboxyl group.^20^ We also synthesized a triphenylmethyl- (trityl-) capped MPS to compare the thiol ester effect on water solubility. In this report, based on the XPS results, all the protecting groups (Ac-, Boc-, and trityl-) can inhibit the oxidation well for the mercapto group. In the case of Ac- and Boc-capping on MPS, the thiol ester raises the modification rate and water solubility and can easily remove the protecting group by acid solution. In the case of trityl-MPS, there is no existing water solubility and it is difficult to remove trityl- by acid water. (A comparative table is shown in Table S1.)

In this report, we used contact angle measurement, XPS for characterizing the mercapto films on the substrate after capping MPS modification, and AuNPs for tracking the kinetic curve of mercapto films on glass under methanol solution and plastic substrate under water; we also used AuNPs to track the ambient stability of the films. Furthermore, we explored the use of Ac-MPS as an adhesive film for anchoring gold nanoparticles on silica surfaces and compared its performance to that of H-MPS. We also compared the sensitivity of LSPR under different substrates and different mercapto films with/without the capping group of MPS. This showed that the LSPR on the plastic substrate has great sensitivity compared to that on the glass substrate in the refraction index test. This creates a new choice of material substrate for biosensors.

## Materials and Methods

### Materials and Reagents

The following chemicals were purchase from different agencies: dichloromethane (ECHO CHEMICAL, HPLC, 99%), toluene (ECHO CHEMICAL, HPLC, 99%), ethanol (ECHO CHEMICAL, anhydrous 99%), dimethyl sulfoxide (SCHARLAB, HPLC, 99%), acetonitrile (ACETONITRILE, HPLC, 99%), sodium bicarbonate (First Chemical, ≥99.7%), sodium carbonate anhydrous (First Chemical, ≥99.9%), bovine serum albumin (SIGMA, heat shock fraction, pH 7, ≥98%), Tween 20 (First Chemical, 0.5 L, 98%), phosphate-buffered saline (Corning, 1×, 500 mL), acetic anhydride (99%, Sigma Aldrich), trityl chloride (ACOS, 99%), (3-mercaptopropyl) trimethoxysilane (Sigma-Aldrich, 95%), di-*tert*-butyl dicarbonate (Sigma-Aldrich, 98%), 2-hydroxyethyl disulfide (Sigma-Aldrich, technical grade, 98%), hydrogen tetrachloroaurate­(III) (Alfa Aesar, solution, Au 40–44% w/w, 1 G), sodium citrate (Alfa Aesar, 99.0%), trimethylamine (Sigma-Aldrich, ≥99.5%), methanesulfonyl chloride (Sigma-Aldrich, ≥98%), dimethylformamide (Alfa Aesar, 99%), and sodium hydroxide (NIHON SHIYAKU REAGENT, 95%). All aqueous solutions were prepared with water that was purified using a Millipore Milli-Q water purification system (Millipore) with a specific resistance of 18.2 MΩ cm. ^1^H (200 MHz) NMR spectra were recorded in DMSO-d6 solution on a Bruker Avance II 400 NMR spectrophotometer. Chemical shifts were reported as positive downfield shifts in ppm, relative to tetramethyl silane.

### Capping MPS Synthesis

Capping MPS was prepared using a similar organic synthetic method of MPS reported earlier. A solution of 196 mg of MPTMS (1 mmol), capping reagent (0.8 mmol) (di-*t*-butyl dicarbonate; 174.4 mg, acetic anhydride; 102 mg; or trityl chloride; 278.8 mg), and 1 g of sodium bicarbonate (11.49 mmol) in 50 mL of acetonitrile was refluxed at 40 °C with stirring in a round-bottom flask for about 6 h. This reaction was monitored by thin-layer chromatography. Then, the powder was removed by the filter (the product was washed with *n*-pentane) and dried with a rotor evaporator to remove the organic solvent. The product was redissolved in 80 mL of dichloromethane (DCM) and recrystallized three times with 10% sodium bicarbonate solution. A transparent oily product of capping MPTMS (92%) was obtained.

A solution of capping MPTMS (1 mmol) and 119.2 mg of triethanolamine (0.8 mmol) in 50 mL of toluene was refluxed with stirring in a round-bottom flask for about 12 h, and a rotary evaporator was used to remove the toluene. Then, the compound was recrystallized by DCM/propane (1:1, v/v) at 4 °C three times. A white solid of Boc-MPS was acquired (yield 74% for Ac-MPS, 80% for Boc-MPS, 65% for trityl-MPS), and finally, DMSO was dissolved as a stock solution and stored at room temperature.

NMR: Boc-MPS: NMR (200 MHz, *d*-DMSO): 0.54 (2H, t), 1.54 (9H, s), 1.64 (2H, m), 2.37 (6H, t), 3.32 (2H, t), 4.02 (6H,t).

NMR: Ac-MPS: NMR (200 MHz, *d*-DMSO): 0.55 (2H, t), 1.63 (2H, m), 2.3 (3H, s), 2.38 (6H, t), 3.31 (2H, t), 4.03 (6H,t).

NMR: trityl-MPS: NMR (200 MHz, *d*-DMSO): 0.53 (2H, t), 1.62 (2H, m), 2.37 (6H, t), 3.31 (2H, t), 4.02 (6H,t), 7.15–7.31 (15H, m).

### Preparation of Gold Nanoparticles (AuNPs)

Gold nanoparticles (AuNPs) were synthesized with the Turkevich method. 20 mL of HAuCl_4_ solution was heated to ebullition in a round-bottom flask, then added with 2.4 mL of sodium citrate solution (1%) with mixing until the color changed to wine red, and cooled to room temperature. AuNP solutions were characterized by UV–visible spectroscopy (Jasco V-570 spectrophotometer), as shown in the Supporting Information. Transmission electron microscopy (TEM) images were acquired, showing a mean diameter of AuNPs of 13 ± 0.3 nm. Then, the AuNP solution was diluted with distilled (DI) water to an absorbance of 2.0 and stored at 4 °C for experiments afterward (data shown in Figure S1).

### Mercapto Film Modification on Silicon Wafer

#### Capping Mercapto Film Modification on Silicon Wafer by Capping MPS

The capping-MPS (Boc-, Ac-, and trityl-MPS) modified substrates under organic solvent were prepared as follows. The stock capping-MPS solution was diluted to 1:1000 (v/v, about 0.46 mM) in toluene, and silicon wafer slides were immersed in the solution for 30 min. Subsequently, the modified glass slides were rinsed with methanol and purified water and then dried by a nitrogen stream.

#### Mercapto Film Modification on Silicon Wafer by MPTMS

The MPTMS modified substrates under organic solvent were prepared as follows. The MPTMS prepared (2%, v/v) in toluene and the silicon wafer slides were immersed in the solution for 12 h. Subsequently, the modified glass slides were rinsed with methanol and purified water and then dried by a nitrogen stream.

### Kinetics of MPTMS and Ac-MPS Modification on Glass under Toluene and Methanol Solvents

#### Ac-MPS Modification on Glass under Toluene or Methanol Solution

The Ac-MPS modified substrates under organic solvent were prepared as follows. The stock Ac-MPS solution was diluted to 1:1000 (v/v, about 0.46 mM) in methanol or toluene, and the glass slides were immersed in the solution for different times (0.5, 0.8, 1, 3, 5, 8, 10, 15, 20, 30, 40 min). Subsequently, the modified glass slides were rinsed with methanol and purified water and then dried by a nitrogen stream.

Glass slides with Boc-MPS modification were immersed in AuNP solution (absorbance is 1.0 at 520 nm) for 30 min. Subsequently, the modified glass slides were rinsed with purified water and then dried by a nitrogen stream.

#### MPTMS Modification on Glass under Toluene or Methanol Solution

The MPTMS modified substrates under organic solvent were prepared as follows. 2% (w/w) MPTMS was prepared in methanol or toluene, and the glass slides were immersed in the solution for different times (1, 3, 5, 7, 10, 30, 50 70, 90, 100, 200, 300, 500, 700, 900, 1000, 1200, 1400 min). Subsequently, the modified glass slides were rinsed with methanol and purified water and then dried by a nitrogen stream.

Glass slides with Boc-MPS modification were put into AuNPs solution (Absorbance is 1.0 at 520 nm) for 30 min. Subsequently, the modified glass slides were rinsed with purified water and then dried by a nitrogen stream.

### Kinetic Curve of AC-MPS Modification on the Plastic Cuvette Surface under Aqueous Conditions

#### Ac-MPS Modification on the Plastic Cuvette under DI Water

The Ac-MPS modified substrates under water were prepared as follows. The stock Ac-MPS solution was diluted to 1:1000 (v/v, about 0.46 mM) in DI water, and the plastic slides were immersed in the solution for different times (0.5, 0.8, 1, 3, 5, 8, 10, 15, 20, 30, 40 min). Subsequently, the modified plastic cuvettes were rinsed with purified water and then dried by a nitrogen stream.

The plastic cuvette with Ac-MPS modification was immersed in the AuNP solution (absorbance is 1.0 at 520 nm) for 30 min. Subsequently, the modified glass slides were rinsed with purified water and then dried by a nitrogen stream.

### Oxygen Plasma

The glass and plastic substrates were treated with oxygen plasma to clean and activate the surface by a plasma cleaner (PDC-001, Harrick Plasma Cleaner). After the pressure was stabilized in the chamber (under 8.3 × 10^–1^ Torr), the capacity coupled RF-discharge plasma was generated using an RF frequency generator. The RF generator was operated at the standard industrial frequency of 13.56 MHz and the controllable nominal power up to 30 W. When O_2_ plasma was introduced to the chamber, 30 W radio frequency forward power was applied to create plasma.

### X-ray Photoelectron Spectroscopy, XPS

The surface chemical states of the samples were characterized by XPS using a Kratos Axis Ultra DLD spectrometer with a vacuumed Mg/Al achromatic source of 450 W. Each sample was analyzed at a photoelectron takeoff angle of 45°. Peak deconvolution was performed by the software XPSPeak 4.1 after Shirley background and optimized with the Lorentzian–Gaussian function curve fitting. All spectra were referenced to the C (1s) peak of the saturated hydrocarbon aliphatic carbon at 284.8 eV as an internal standard.

The surface structure and morphology of the samples were characterized by XPS using K-alpha X-rays of aluminum with a He I source of 450 W. Peak differentiating and fitting were performed by the software XPSPeak 4.1 after Shirley background and optimized by a fast Fourier transform (FFT) smoothening process. All spectra were calibrated to the C 1s peak at 284.8 eV.

### Scanning Electron Microscopy, SEM

Field emission scanning electron microscopes (FE-SEM, Hitachi SU8020) operated at 15.0 kV for glass slides and 10.0 kV for polystyrene were used to obtain the images of AuNPs on the substrates.

### Ambient Stability Test

The modification of the substrates by MPTMS (toluene, 16 h), MPS (MeOH, 30 min), and Ac-MPS (water, 15 min) was under dark and room temperature conditions according to the procedures described above.

Once a substrate had finished the modification procedures, the stability of the modified substrate was tested immediately under room light and room temperature conditions for a standing time of 3 days. Then, the modified substrate was immersed in a diluted AuNP solution to anchor the AuNPs on the modified surface. The result of the stability test was observed by measuring the UV–visible spectra of AuNPs anchored on the modified substrate.

## Results and Discussion

### X-ray Photoelectron Spectroscopy of Different Silatrane Film Coatings on Glass and Plastic Substrates

XPS is a popular and powerful instrument to measure the atomic states on substrate surfaces.^21,22^ In this report, all XPS signals were calibrated using the 284.8 eV (C–C) peak. Here, the plastic and glass surfaces were cleaned by oxygen plasma, and also, the plastic surface was activated to produce a hydrogen bond for silanization.

In this part, XPS was used to measure the surface modification by different kinds of silatrane molecules. At first, we tested capping MPS (Ac-MPS, Boc-MPS, and trityl-MPS) modification on the glass surface under alcohol (shown in Figure 1A). Second, we modified Boc-MPS and H-MPS on glass and plastic substrates under water (shown in Figure 1B). Third, we measured the effect of conjugation with AuNPs by Ac-, Boc-, and trityl-mercapto films on glass (shown in Figure 1C) and characterized the Ac- and Boc-mercapto films on plastic.

**Figure 1:**
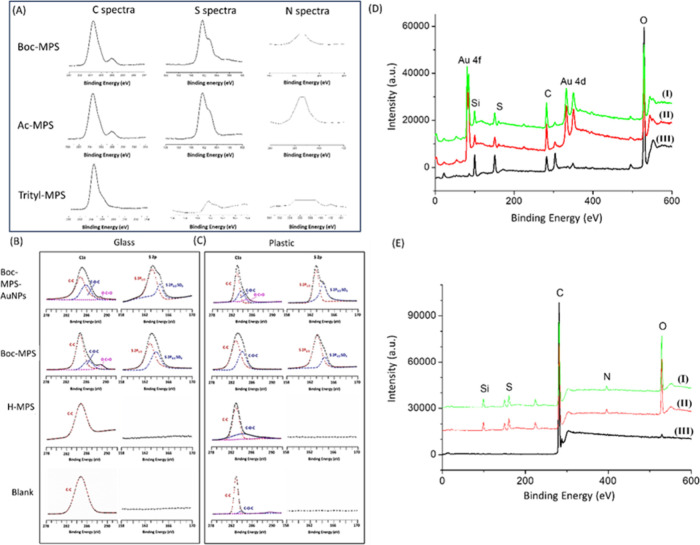
XPS results of the carbon, thiol, and nitrogen atom spectra of Boc-MPS, Ac-MPS, and trityl-MPS group capping MPS coating on the glass substrate under 75% alcohol solution. (A). XPS spectra of C 1s and S 2p. (Lower) Glass and plastic substrates without modification. (Middle) Glass and plastic substrates modified with Boc-MPS in DI water, (upper) Au-NPs anchored on the surface of glass (B), and the plastic (C) substrate modified with Boc-MPS in DI water. The XPS spectra of AuNPs anchored on the glass substrate by Ac-MPS (I), Boc-MPS (II), and trityl-MPS (III) (D). The XPS of bare plastic (III), Ac-MPS (I), and Boc-MPS (II) modification on the plastic substrate under water (E).

First, we modified the Ac-, Boc-, and trityl-mercapto films on the glass surface though Ac-MPS, Boc-MPS, and trityl-MPS under 75% alcohol solution and used XPS for characterization of the mercapto films on glass. Data are shown in Figure 1A. In the C spectra of XPS, there is a significant signal at 288.8 eV, attributed to the carboxyl group (O–C=O) from the thiol ester group in Ac- and Boc-MPS.^23^ This special peak showed the successful Ac- and Boc-MPS modification on the glass substrate. In the S spectra of XPS, we can find significant special thiol atom signals in 161.9 and 163.4 eV on the substrate surface after Ac-, Boc-, and trityl-MPS coating.^24^ These two peaks also show that the thiol films successfully inhibit the oxygen oxidation by the protecting group. When we removed the protective group of Ac- and Boc- by 2 M citric acid solution, the S atom shows a thiol oxidation state like the MPS coating.^13^ This is also proven by the fact that the protecting group can successfully inhibit the thiol oxidation. We also tried to use an acid solution like citric acid or trifluoroacetic acid to remove the trityl protecting group on mercapto films. It is interesting that the trityl is not easily removed by acid solution (data not shown). In the N spectra of XPS, we can find a visible peak of the triamino group in the mercapto films of Ac- and Boc-MPS.^25,26,42^ Probably, the Ac- and Boc-MPS have a hydrogen bond donor group interacting with the hydrogen bond acceptor H atom from the triethanol amine. The trityl group without a hydrogen bond donor will not interact with triethyl amine.

Second, we also used XPS to measure the capping effects to MPS and H-MPS modification on glass under water. We modified Boc-MPS and H-MPS on a glass surface under pure water, and we used Boc-MPS and H-MPS modification on glass and plastic substrates under a water environment and used XPS to characterize the mercapto films of Boc-MPS-derived and MPS-derived films on plastic and glass substrates. Here, XPS was also used for characterizing the glass and plastic substrates conjugated with AuNPs by Boc-MPS coating. Data are shown in Figure 1B,C.

The XPS results of the glass substrate without any coating (the blank substrate) show no significant peaks at S 2p and an absorbance peak at the C 1s spectrum for background noise (as shown in the Figure 1B,C). In the XPS result of the glass/plastic substrate with MPS modification under water, the result is similar to that of the blank. In the S 2p spectrum, there is no significant signal from the glass/plastic substrate, showing that the MPS is not successfully modified on the glass/plastic surface under a water environment. In the XPS result of the glass substrate with Boc-MPS modification under water, there are significant peaks detected in the S 2p spectra such as 161.9 (S3/2) and 163.4 (S1/2) eV. In the C 1s spectrum, fitted by the XPSPeak program, several peaks are found at 284.8 (sp3 C–C), 286.2 (C–O–C) eV, and 288.8 eV (sp2 O–C=O). In the XPS result of the plastic substrate with Boc-MPS modification under water, the spectra of S 2p and C 1s both have significant signals from Boc-MPS. In the C 1s spectrum, the 288.8 eV signal is decreased and not easily found; that from the carbon signal for plastic is stronger than the carboxyl group of Boc-, and for this reason, the carboxyl signal is not so clear here.

In this report, this is the first time that the Ac- and Boc- of the silatrane molecular can be modified on a substrate under a water solution. This is a huge breakthrough in the silane molecular studies. The organic solvent necessary for silanization is limited for the material of the substrate; the substrate usually needs to be a metal, silicon wafer, or glass. The base on the capping MPS can dissolve in water and modification substrate under water, and the applicability of capping MPS can be extended to plastic. The protecting group of Boc- successfully increases the water solubility of Boc-MPS, but when the concentration increases to 1 mM, the Boc-MPS water solution will produce some precipitation in the solution, which means that the thiol ester group can raise the solubility of Boc-MPS, but not too much. However, Boc-MPS only needs 0.4 mM for finishing the surface modification on the substrate. In our experience, polyethylene (PE), polypropylene (PP), and polystyrene (PS) plastics can be modified by Boc-MPS under water.

Third, the glass surface can conjugate with AuNPs though mercapto films from Boc-MPS. There is a strong Au 4f peak in the XPS results in Figure 1D. The results show that the Boc-MPS can successfully modify a glass substrate under a water environment.

In Figure 1D, XPS was also used to carefully characterize the AuNPs anchored on the glass surface by Boc-, Ac-, and trityl-capped mercapto films, with the data shown in Figure 4C. In Figure 4C, the AuNPs were anchored by different capping MPS modifications on the glass substrate (Ac-MPS and Boc-MPS modification on the substrate under water and trityl-MPS modification under 75% alcohol). In the full spectra, mercapto films were conjugated with AuNPs from Ac-MPS (I, green), Boc-MPS (II, red), and trityl-MPS (III, black). In the XPS spectra, we can find significant Au peaks in the mercapto films from Ac- and Boc-MPS, meaning that the thiol ester group of Ac- (green line; I) and Boc-MPS (red line; II) can bind with AuNPs. However, in the case of trityl-MPS, there is no significant Au signal in XPS, showing that the trityl capping mercapto films cannot bind with AuNPs. Compared to the result of trityl-MPS in Figure 1A, the trityl group successfully inhibited the oxidation of the thiol group in trityl-MPS, but the trityl also restrained the effect of thiol conjugated with AuNPs.

In Figure 1E, we used XPS to measure the mercapto film modification on the plastic substrate by Boc-MPS and Ac-MPS. The XPS spectra show the bare plastic (black; line III), the Ac-MPS coating (red; line II), and the Boc-MPS coating (green; line I) on the plastic surface. The black line in Figure 1E, based on the substrate, is bare plastic: there is a significant signal of the carbon atom found, but there are no signals of Si, N, and S atoms found. The red and green lines are shown in Figure 1E. Apart from the visible carbon atom signal found, there are significant signals of Si, N, and S atoms also. In the S spectra, there shows a reduction thiol group, proving that the thiol ester group can protect the mercapto from oxidation by oxygen. In the N spectra, we also find a signal from triethanolamine. Just like the hypothesis before, the thiol ester also traps the triethanolamine on the substrate surface. However, this result shows that the Ac-MPS and Boc-MPS both can be modified on the plastic surface under water. This is the first time that using capping silatrane was a success for creating mercapto films under water.

### Surface Hydrophilic Test of Different Silatrane Films on the Glass Surface through the Water Contact Angle

Contact angle is commonly used for measuring the hydrophilic and hydrophobic effects on substrate surfaces. In this report, we used the water contact angle to further characterize the silane molecular (MPTMS, Ac-MPS, Boc-MPS, and trityl-MPS) modification on silica wafer. Data are shown in Figure 2A. The contact angle for oxygen plasma and ethanol cleaned silicon wafers was 25.1° ± 3.3°, which is indicative of a hydrophilic surface. The contact angle for the MPTMS and capping-MPS created mercapto films was much higher, at 56.4° ± 2.1°, and that for the Boc-group was 69.4° ± 4.2°, that for the Ac-group was 65.3° ± 2.2°, and that for the trityl-group was 94.6° ± 4.5°. The Boc-, Ac-, and trityl- mercapto films created a hydrophobic structure on silicon wafers; especially, in the trityl group, the multibenzyl group shows a strong hydrophobic effect of mercapto films on silicon wafer. The contact angle for the capping-MPS films was 55.3° ± 1.1° for Ac- and 56.1° ± 2.3° for Boc- after 2 M citric acid treatment, which shows that the acid solution can easily remove the protecting groups Ac- and Boc-. Here, in the case of trityl-MPS films on silicon wafer, we also used citric acid and 3 M trifluoroacetic acid (TFA) to remove the trityl group of mercapto films. However, the contact angle was still higher than 90° after acid solution treatment for 30 min, showing that the trityl group is not easily removed by acid solution, and this effect limits the functional application of trityl-MPS in anchored metal nanoparticle. (Data are shown in Figure S2.)

**Figure 2:**
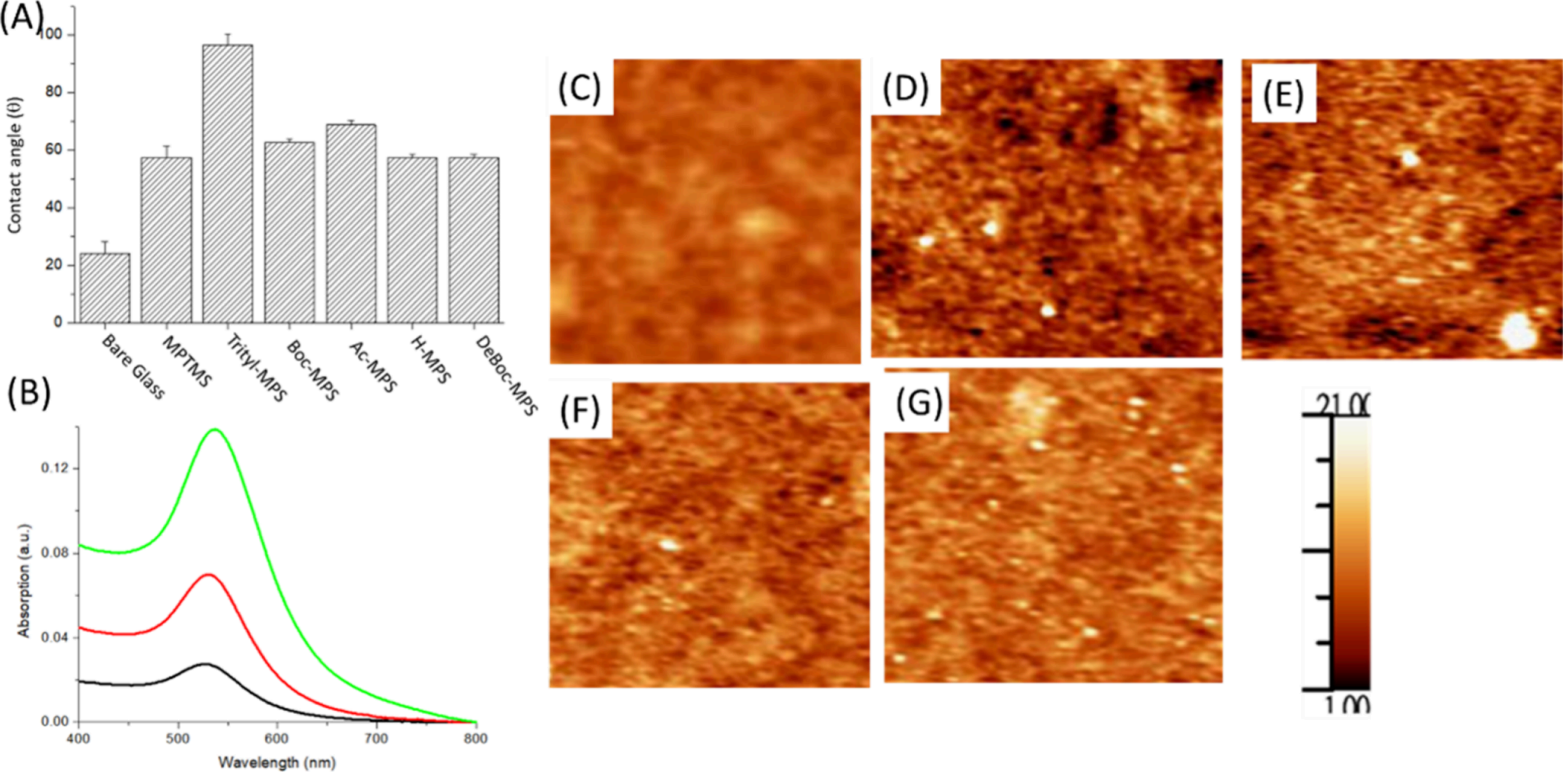
Contact angles of bare glass, MPTMS, Ac-MPS, Boc-MPS, trityl-MPS, and H-MPS modified on glass and the contact angle after 2 M citric acid solution treatment for glass with Boc-MPS coating (A). The UV–visible spectra of MPTMS, H-MPS, and Ac-MPS coated glass slides were exposed in ambient air under room light at 7 days and then immersed in a solution of AuNPs for 30 min (B). The AFM images of bare glass (C), MPTMS (D), Boc-MPTMS (E), Boc-MPS (F), and trityl-MPS (G) coating on the glass substrate.

### Ambient Stability Test

The stability of mercapto films on the surface is a very important issue in anchoring metal nanoparticles on the substrate. Here, we used AuNPs to track the stability of mercapto films.^13^ We tested the stability of mercapto films from Ac-MPS, MPS, and MPTMS under an atmosphere environment. Data are shown in Figure 2B.

The stability test of the modified substrate was performed under room light and room temperature conditions for a standing time of 3 days and tracked the functional property of mercapto films by AuNPs. The result of the stability test was observed by measuring the UV–visible spectra of the AuNPs anchored on the modified substrate.

We can find that the absorbance of AuNPs anchored on the substrate by MPTMS is lower than those by MPS and Ac-MPS. This means that the mercapto films are already losing the functional binding with the gold nanoparticles. In the case of Ac-MPS anchored AuNPs, it still has a high effect for binding with AuNPs, and the absorbance is around 0.13. It has more than 95% functional binding with AuNPs (the start of the maximum of AuNP absorbance is 0.135, data not shown). In the case of MPS anchored AuNPs, we can find that the mercapto films still have 60% of the functional binding after 3 days.

### Measuring the Shape of Films on Glass by Different Silanes/Silatranes through AFM

Atomic force microscopy (AFM) is a common instrument used for measuring the substrate states and the structures on substrates.^27,28^ Figure 2C–G shows a typical series of AFM 2D images displaying the mercapto films on the glass substrate.

The size of the AFM scans is 1 × 1 mm^2^. Figure 2C shows a bare glass surface without any coating, and the surface roughness is 316 pm. This shows a smooth surface under the AFM image. The substrate shows a smooth surface after mercapto films are coated by MPTMS (Figure 2D) and Boc-MPTMS (Figure 2E). The surface roughness is increased to 462 pm for MPTMS and 569 pm for Boc-MPTMS mercapto films, and few small protrusions appear on the surface of MPTMS and Boc-MPTMS coating. The bright spots in the image indicate where aggregates have formed. Such an observation is consistent with previous reports of MPTMS and is attributed to the competition between self-polymerization and condensation reaction with surface silanol.^13^ The higher roughness of the MPTMS film has been attributed to unhydrolyzed Si–OCH_3_ bonds, which make lateral cross-linking between neighboring adsorbed MPTMS molecules more difficult.^13^ Incomplete lateral cross-linking may result in lower surface coverage.

Figure 2F,G shows the mercapto films of Boc-MPS and trityl-MPS, respectively. Both mercapto films have a smooth surface after coating. The surface roughness is reduced to 198 pm for MPTMS and 241 pm for Boc-MPTMS mercapto films. This shows that the silatrane is helpful for silane assembly on the substrate surface.

### Kinetic Curves of Boc-MPS/MPTMS Modification on Glass

The mercapto group commonly functions as an anchor to noble metal nanoparticle. For this, we can use the AuNPs to track the mercapto film structure on the substrate. Here, 20 nm AuNPs were used to track the mercapto films on the substrate, and the AuNPs show a special absorbance peak at 520 nm in the UV–visible spectrum.

The polarity of solvent will affect the silanization of silane molecular modification on the substrate surface.^29–32^ In this report, we measure the kinetic curves of MPTMS and Boc-MPS modification on the glass substrate under different organic solvents (toluene and methanol), with the data in Figure 3A,B. All the results were measured and calculated at 520 nm. In Figure 4A, the kinetic curve of MPTMS modification on the glass surface is different under toluene (black square) and methanol (red circle). The MPTMS needs to spend around 400 min to provide high cover mercapto films on the glass surface, but the high cover mercapto films need more than 1000 min under methanol. Compared with previous data, the nonpolar organic solvent, toluene, is beneficial to the grafting reaction.^32,43,44^

**Figure 3:**
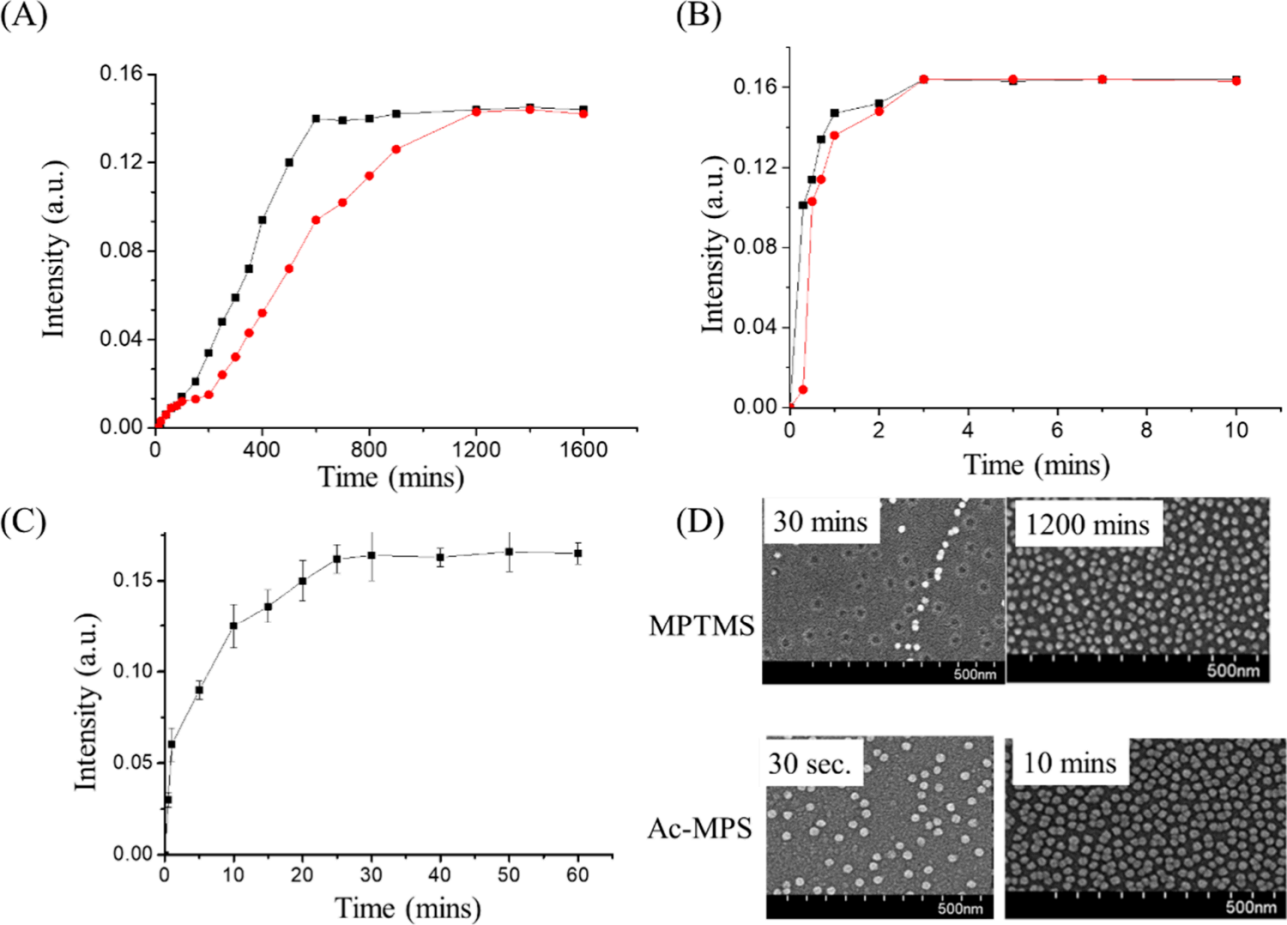
Using AuNPs to track the kinetic curves of MPTMS (A) and Boc-MPS (B) modified on the glass substrate under toluene (black square) and methanol (red circle) organic solvents. The AuNP tracked kinetic curve of Boc-MPS modified on the plastic substrate under water (C). The SEM images of MPTMS and Boc-MPS modification AuNPs on glass under different times (D).

**Figure 4:**
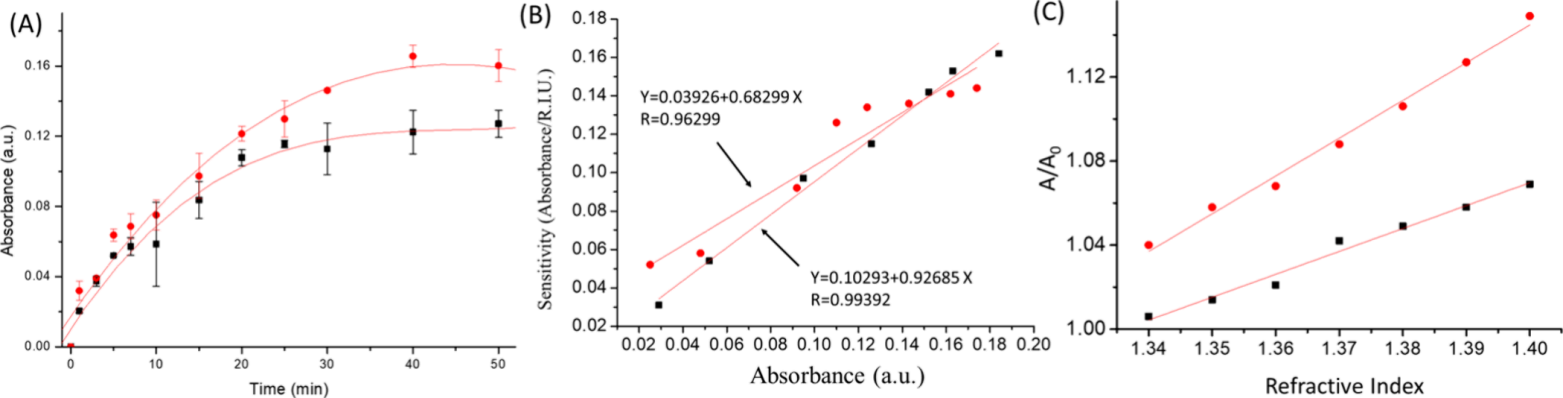
AuNPs anchored on the glass surface by MPTMS (black square) and Boc-MPS (red circle) at different times (A). The relation of sensitivity and AuNP coverage by MPTMS (red circle) and Boc-MPS (black square) anchored AuNPs on the glass surface (B). The sensitivity of LSPR refractive index test (C) on glass (black) and plastic (red) substrates.

The result of Boc-MPS modification on the glass surface under toluene (black squire) and methanol (red circle) is shown in Figure 4B. In Figure 4B, the result is totally different from that of MPTMS. There is no significant difference between the kinetic curves of Boc-MPS modification on the glass surface under toluene and methanol. Boc-MPS requires only 4 min to form a complete layer with the optimum mercaptan surface density.

Figure 3C shows the kinetic curve of Boc-MPS modified on a plastic surface under a water environment. In the data, the Boc-MPS maximum modification on the plastic surface is 30 min and the absorbance of AuNPs at 520 nm is around 0.16 (a.u.).

### SEM of AuNP Coating on Glass by MPTMS and Boc-MPS

Scanning electron microscopy (SEM) is widely used for imaging the surface.^33,34^ In this report, we also used SEM to characterize the phase morphology of AuNP modification on the glass surface. Figure 4D shows the MPTMS modification on the glass surface after 30 min under toluene and anchored AuNPs after 30 min. We can find that there are some AuNPs modified on the glass surface localized and those AuNPs are close to each other. Together with the AFM image of MPTMS films, the surface has an island structure. The island structure will induce AuNPs localized to anchor on the glass surface. When the time was increased to 1200 min of MPTMS modification on glass, the AuNPs become uniform and provide high coverage on the glass surface.

Figure 4D shows the AuNP layer on mercapto films by Boc-MPS modification in 30 s. The AuNPs have uniform dispersion on the glass surface, and when the time of modification is increased to 10 min, the AuNPs become uniform and have high coverage on the glass surface.

### Kinetic Curve of AuNPs Anchored on Glass through Mercapto Films from MPTMS and Boc-MPS

Figure 4A shows the kinetic curve of AuNPs anchored on mercapto films. The AuNPs requires around 35 min to form a complete AuNP layer with optimum mercaptan surface density anchored on MPTMS (black square) and Boc-MPS (red circle) films. The result shows that there is no significant difference in mercapto films binding with AuNPs of MPTMS and Boc-MPS films. This shows that the fresh mercapto films from MPTMS have a good effect of binding AuNPs, compared with the data in Figure 2B. The mercapto films from MPTMS will lose the effect of conjugation with AuNPs under a light room environment after 7 days. The mercapto films from Boc-MPS show a similar effect of conjugated AuNPs in fresh preparation of mercapto films on glass; compared to Figure 2B, Boc-MPS still shows an excellent effect of binding with AuNPs under a light room environment after 7 days. This result shows that the Boc- group successfully increases the stability of mercapto films.

### Relation of Sensitivity by Coating AuNPs with Ac-MPS on Glass and Plastic

AuNPs have a unique local surface plasmon resonance (LSPR) effect and are widely used in biosensor design.^35^ Sucrose solution in different concentrations was used in the refractive index test of the LSPR biosensor test.^36–38^ The refractive index test simply shows the sensitivity of the biosensor system.^39–41^

In this report, we also used the refractive index test to measure the sensitivity of the LSPR effect under different AuNP coverages by MPTMS and Boc-MPS coating. The slope of the refractive index test was defined as sensitivity in different AuNP coverages by MPTMS (red circle) and Boc-MPS (black square) on the glass surface. The coverage of AuNPs on the glass surface and, at that coverage, the sensitivity to the refractive index of sugar solutions at different concentrations are plotted. At similar absorbance of AuNPs on the glass surface, the sensitivity of MPTMS coating AuNPs is better than that of Boc-MPS under low AuNP coverage, but with increasing coverage of the AuNP coverage, the sensitivity of AuNPs anchored by Boc-MPS is better than that by MPTMS.

The refractive index test showed the AuNPs anchored on glass (Figure 4C - black) and plastic (Figure 4C - red) by Boc-MPS. Under similar AuNP absorbance at 520 nm, the LSPR has higher sensitivity of AuNP modification on plastic than on the glass substrate in the refractive index test. This result shows a new substrate for LSPR biosensors.

## Conclusions

In this study, we have shown that capping MPS can be used to form a self-assembled film on silica surfaces and succeeded for inhibiting the oxidation by oxygen. XPS, contact angle measurement, and AFM are applied to measure the MPTMS, MPS, and capping MPS modification on the substrate. All the functional parameters are shown in Table 1. In some cases, thiol ester protecting groups such as Ac-MPS and Boc-MPS also act as effective adhesive layers for the construction of a gold colloid monolayer on silica surfaces, as silatranes are relatively stable to moisture. In comparison with MPTMS, the Boc-MPS films are smoother and more uniform and are completely free of molecular aggregates. Boc-MPS is water-soluble and can be modified under water, while MPTMS usually works better in anhydrous organic solvents. This shows that the cost of Boc-MPS is lower than that of MPTMS, as shown in Table 2. Boc-MPS also requires a significantly shorter time than MPTMS to form a complete layer and exhibits a higher mercaptan surface density in the film. As a result, the saturation coverage of the gold colloid monolayer on the Boc-MPS coated substrates is higher than that on the MPTMS-coated substrates. Surprisingly, the Boc-MPS films also exhibit a higher ambient stability compared to the MPTMS films. This characteristic is beneficial to many applications where the sufficient durability of the self-assembled films under ambient conditions is important. Thus, Boc-MPS as an efficient and environmentally amiable molecular linker may have a potential to replace MPTMS or MPS for surface modification. In the LSPR sensitivity of the refractive index test, the mercapto film-anchored AuNPs have better sensitivity from Boc-MPS than MPTMS. Interesting is that the LSPR sensitivity of the refractive index test on plastic is better than that on the glass substrate. This also introduces a new perspective for selecting novel substrates for the development of future biosensors.

**Table 1:** Comparison of MPTMS, MPS, Ac-MPS, Boc-MPS, and Trityl-MPS Surface Modification

parameter	**MPTMS**	**H-MPS**	**Ac-MPS**	**Boc-MPS**	**trityl-MPS**
molarity used in surface modification	0.1 M	1 mM	0.24 mM	0.24 mM	0.24 mM
time for surface modification	500 min	30 min	5 min	5 min	5 min
solvent used in surface modification	organic solvent	organic solvent/alcohol solution	water	water	organic solvent/alcohol solution
water solubility	no	no	yes	yes	no
plastic modification	no	no	yes	yes	no
mercapto film oxidation under ambient	oxidation	oxidation	no oxidation	no oxidation	no oxidation
the effect of conjugated AuNPs	yes	yes	yes	yes	no
cost of surface modification^a^	4.84 USD	1.91 USD	0.288 USD	0.312 USD	0.957 USD

a Means the cost of each 100 mL of solution applied to surface modification with MPTMS, H-MPS, Ac-MPS, Boc-MPS, and trityl-MPS as shown in Table 2.

**Table 2:** Cost Table of Each 100 mL Solution Applied to Surface Modification with MPTMS, H-MPS, Ac-MPS, Boc-MPS, and Trityl-MPS^a^

**parameter**	**MPTMS**	**H-MPS**	**Ac-MPS**	**Boc-MPS**	**Trityl-MPS**
price per gram	1.98 USD	3.8 USD	4.1 USD	3.7 USD	6 USD
solvent (per 100 mL)	0.88 USD (toluene)	0.25 USD (75% ethanol)	0.003 USD (water)	0.003 USD (water)	0.25 USD (75% ethanol)
molarity used for surface modification	0.1 M	1 mM	0.24 mM	0.24 mM	0.24 mM
cost of surface modification (Solution for 100 mL)	4.84 USD	1.91 USD	0.288 USD	0.312 USD	0.957 USD

a Each kind of silane/silatrane molecular has a different molarity and solvent for preparing the solution for surface modification.

In this report, the new idea of capping effects gives a new model of environmentally friendly structure of silatrane; this new model successfully created a new choice of design water-stable silane molecular theory. This model can be widely applied for glass/plastic biosensor surface modification, nanoparticles anchored on the substrate, and biomaterial surface functionalization. The new capping-MPS structure breaks through material limitations. It can be applied to new biosensor platform building, biomaterial modification (conjugated biomimicry structure to raise the biocompatibility of materials or conjugated with drug for drug control release), and sensing for water quality (heavy metal ion detection). With growing environmental awareness in recent years, this approach represents a significant advancement in eliminating the need for organic solvents traditionally required for silane-based surface modification. Instead, it enables the entire process to be conducted in aqueous solutions, aligning fully with environmentally friendly principles.

## Supplementary Material



## Notes

Comparative table of MPTMS, H-MPS, and capping-MPS; contact angles of MPTMS, H-MPS, and capping-MPS on glass (PDF)
