# Supplementary material for: Different Protecting Groups to Cap Mercapto Propyl Silatrane Affect Water Solubility and Surface Modification Efficiency
Source: ACS Omega. 2025 Oct 1;10(40):46343–52. doi: 10.1021/acsomega.4c06255 (PMC12529168; doi:10.1021/acsomega.4c06255)
Supplement: Supplementary file 1 [file ao4c06255_si_001.pdf]

Supporting information.

Different protect group in the capping effect of mercapto propyl silatrane affect the water solubility and surface modification efficiency.

Wen-Hao Chen[af+], Chih-Yu Chen[bc+], Hui-Yin Huang[a], Yu-Cheng Hsiao[def*]

[a.] Research and Development Group, Leo Verification System Inc. Wyoming, US.

[b] Department of Orthopedics, Shuang Ho Hospital, Taipei Medical University, New Taipei City 23561, Taiwan

[c] International Ph.D. Program in Biomedical Engineering, College of Biomedical Engineering, Taipei Medical University, Taipei 11031, Taiwan

[d] Graduate Institute of Biomedical Optomechatronics, College of Biomedical Engineering, Taipei Medical University, Taipei 11031, Taiwan.

[e] School of Biological Sciences, Nanyang Technological University, Singapore 639798, Singapore

[f] Cell Physiology and Molecular Image Research Center, Wan Fang Hospital, Taipei Medical University, Taipei 11031, Taiwan

[+] These authors contributed equally to this work

	MPTMS	H-MPS	Capping-MPS
Moisture sensitivity	Yes	No	No
Surface modify	Slow	Medium	Fast
Water solubility	No	No	Yes
Application	1992	2014	In this report

Table S1. comparative table of MPTMS, H-MPS and capping-MPS.

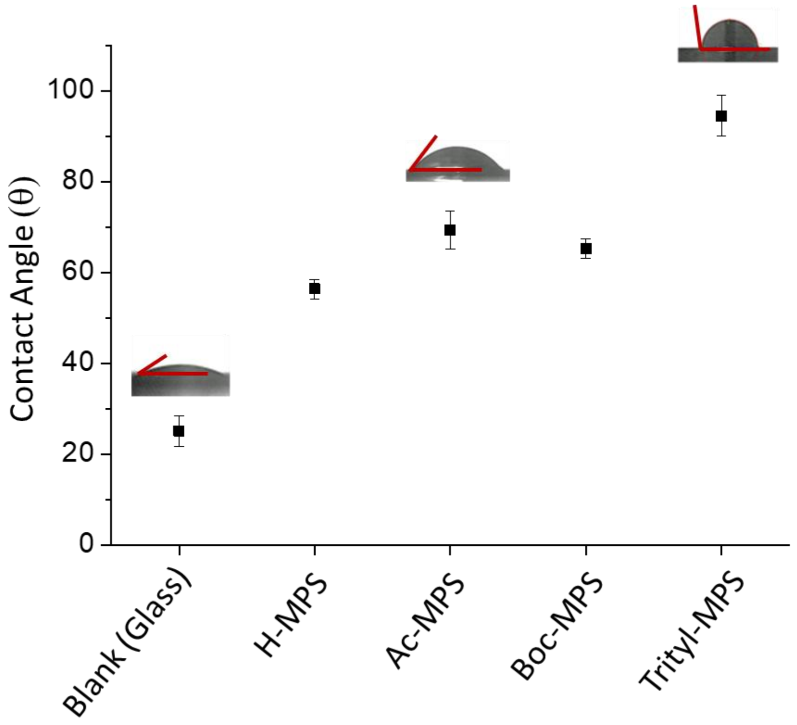

Figure S1. Contact angle of MPTMS, H-MPS and capping-MPS films on glass substrate.
